# Supplementary material for: Integrated Analysis of Metabolome and Transcriptome Provides Insights into Flavonoid Biosynthesis of Pear Flesh (Pyrus pyrifolia)
Source: Foods. 2025 Oct 30;14(21):3716. doi: 10.3390/foods14213716 (PMC12610558; doi:10.3390/foods14213716)
Supplement: Supplementary file 1 [file foods-14-03716-s001.zip › Supplementary Files Materials.pdf]

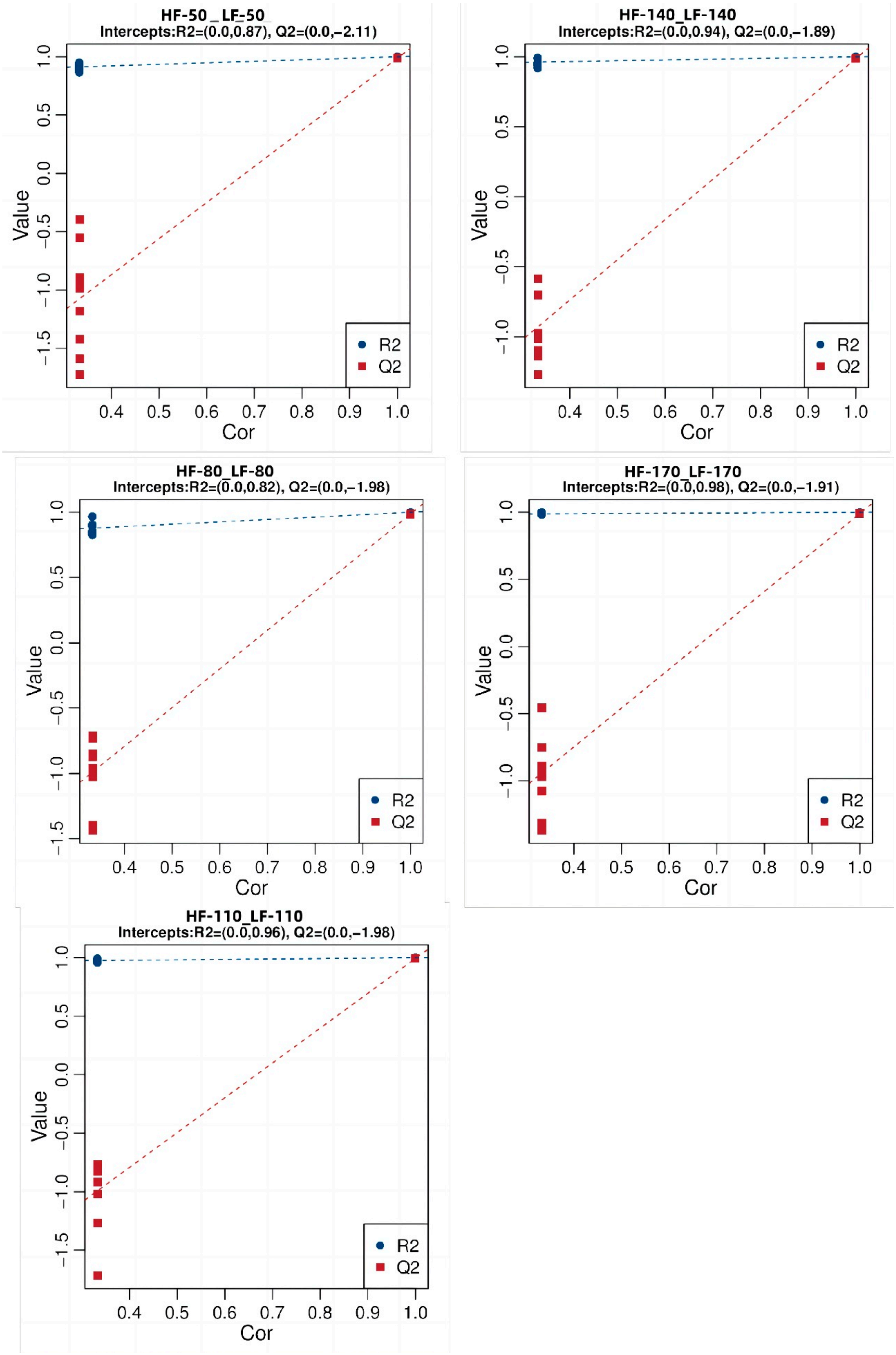

Figure S1. Permutation test (n=200) for the PLS-DA model. The x-axis represents the correlation between randomly grouped Y and the original grouped Y, and the y-axis represents the scores of R^2 and Q^2. The regression lines of R^2 (green) and Q^2 (blue) have intercepts on the y-axis lower than the original model (rightmost point), indicating no overfitting.

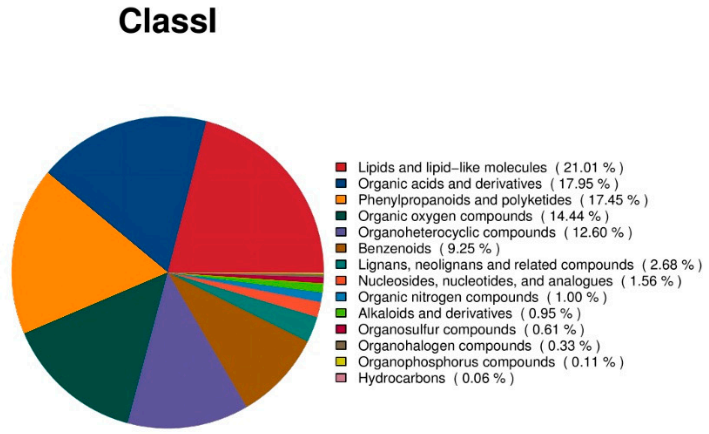

Figure S2. Class distribution of all metabolites detected in the untargeted metabolomics analysis. The name of the classification is given before the parentheses, and the number in the parentheses represents the proportion of that classification in the total number of metabolites.

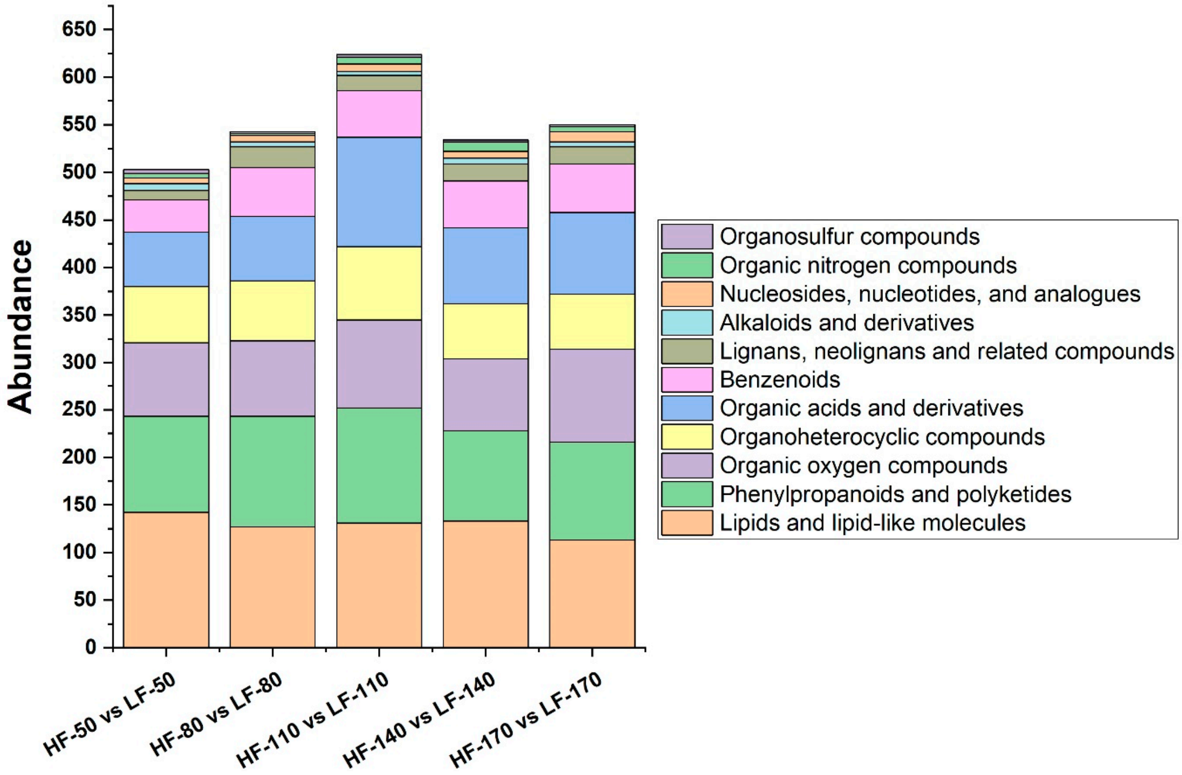

Figure S3. Class composition and quantitative dynamics of different metabolites across five development stages. Labels on the horizontal axis (e.g., "HF-50 vs LF-50") indicate the metabolomic differences between HF and LF pulp at 50 days after flowering (DAF), analogous comparisons were made at 80, 110, 140 and 170 DAF. Differential metabolites were defined as VIP > 1 and $p < 0.05$. The height of each stacked bar represents the total number of different metabolites at the given stage,

and the colored segments correspond to the different superclasses. Lipids and lipid-like molecules remained the predominant classes, followed by Phenylpropanoids and polyketides.

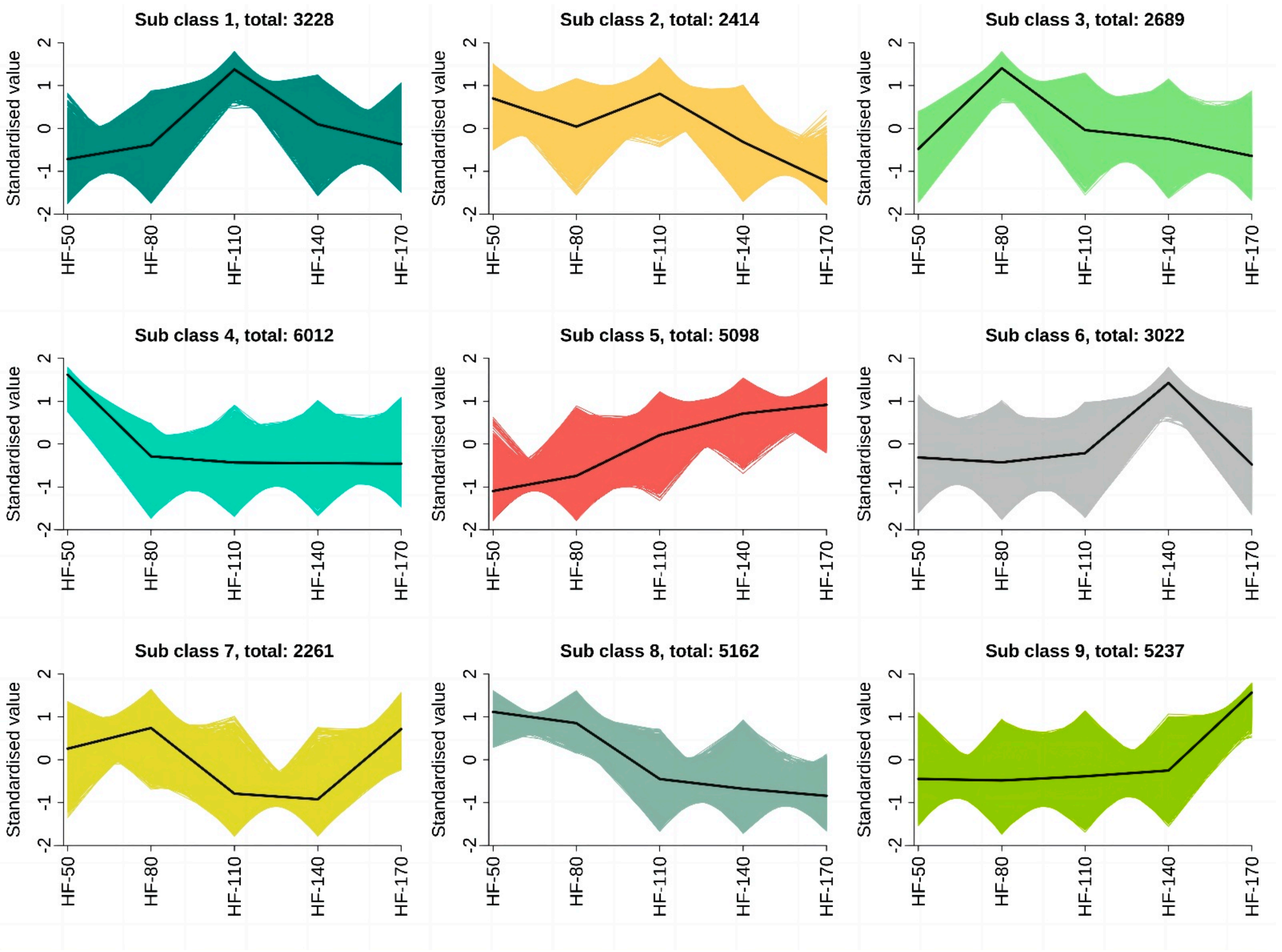

Figure S4. K-means clustering (k = 9) of gene-expression profiles during 'Heqingxiaoshali' pulp development. A total of 35123 genes were normalized (Z-score) and clustered across 5 time points according to their FPKM values. Each panel shows the mean expression trend (solid line) ± SE (shaded area). The x-axis indicates days after flowering (DAF), and the y-axis represents the normalized expression level (Z-score). Curves of the same color represent genes within the same cluster; the total gene number is given in the top-right corner of each subplot.

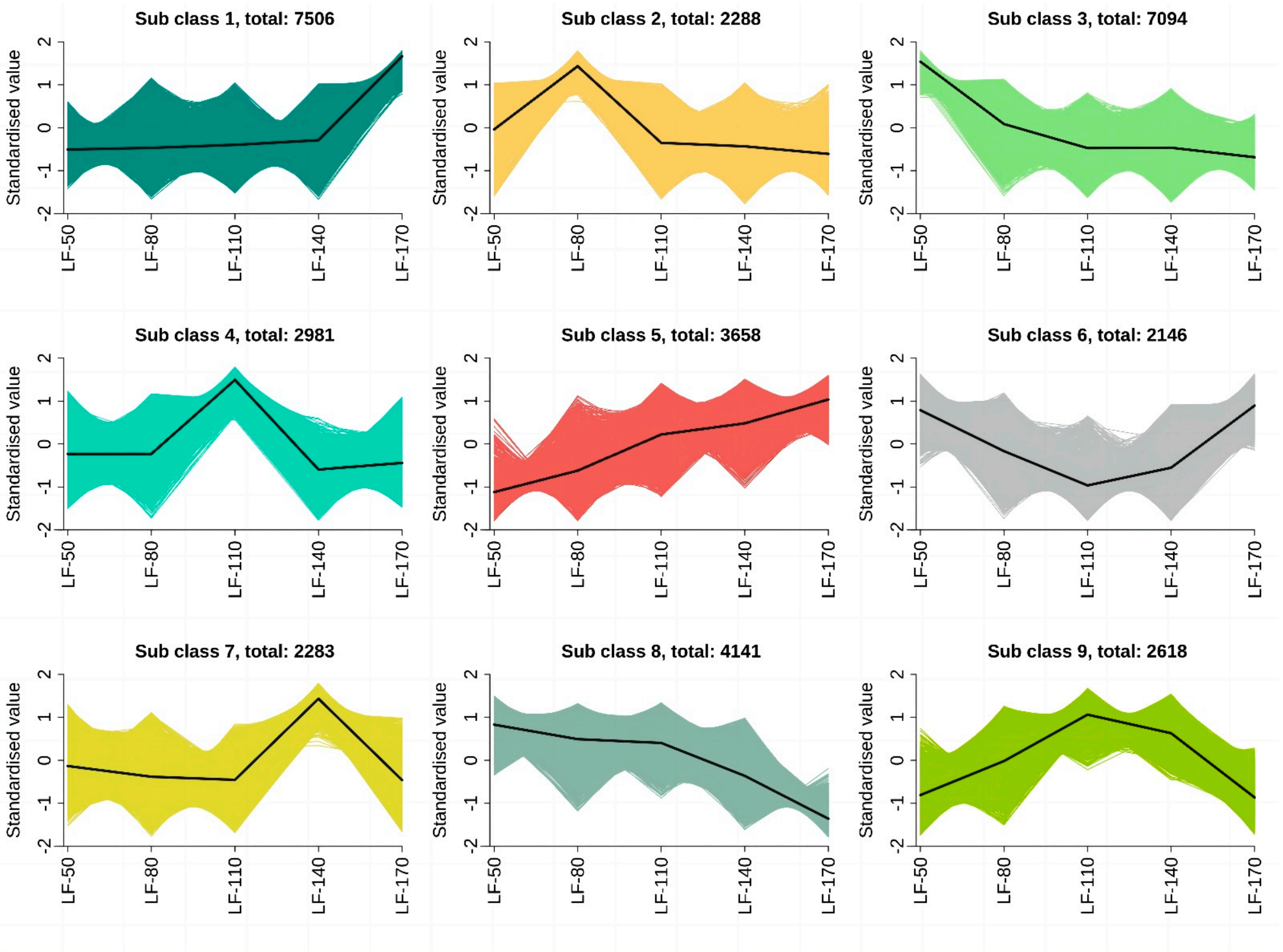

Figure S5. K-means clustering (k = 9) of gene-expression profiles during 'Lunanhuangpili' pulp development. A total of 35123 genes were normalized (Z-score) and clustered across 5 time points according to their FPKM values. Each panel shows the mean expression trend (solid line) ± SE (shaded area). The x-axis indicates days after flowering (DAF), and the y-axis represents the normalized expression level (Z-score). Curves of the same color represent genes within the same cluster; the total gene number is given in the top-right corner of each subplot.

Table S1: The linear equation and correlation coefficient of the standard curve for flavonoid monomers.

Table S2: RT-qPCR primer sequences.

Table S3: Overview of transcriptome sequencing of pulp from ‘Heqingxiaoshali’ and ‘Lunanhuangpili’ at different developmental stages.

Table S4: Alignment and detection of transcriptome sequencing results with the reference genome.
