# Supplementary figures and images for: Integrated Analysis of Metabolome and Transcriptome Provides Insights into Flavonoid Biosynthesis of Pear Flesh (Pyrus pyrifolia)
Source: Foods. 2025 Oct 30;14(21):3716. doi: 10.3390/foods14213716 (PMC12610558; doi:10.3390/foods14213716)

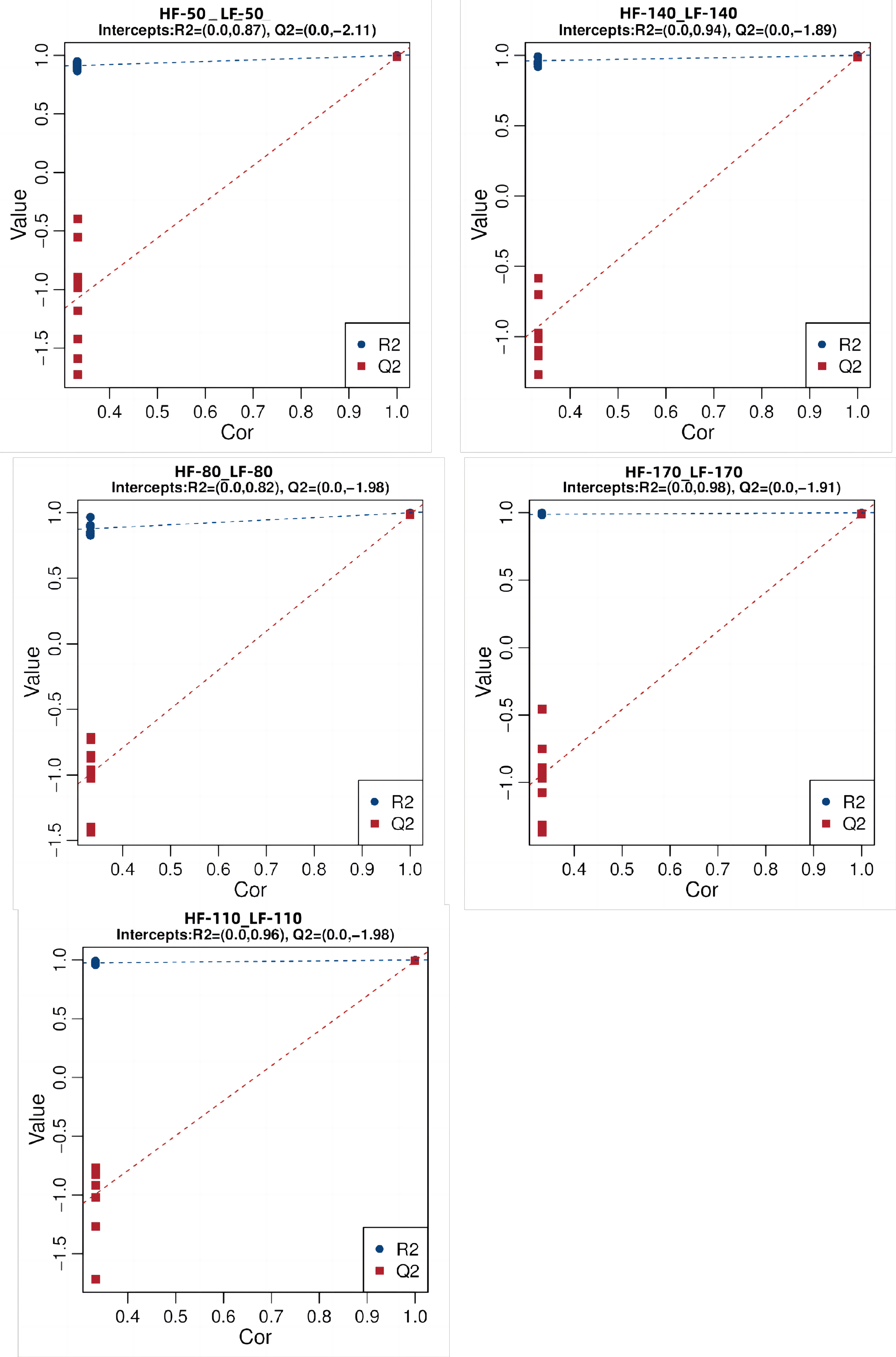

Supplement: Supplementary file 1 [file foods-14-03716-s001.zip › Figure S1.jpg]

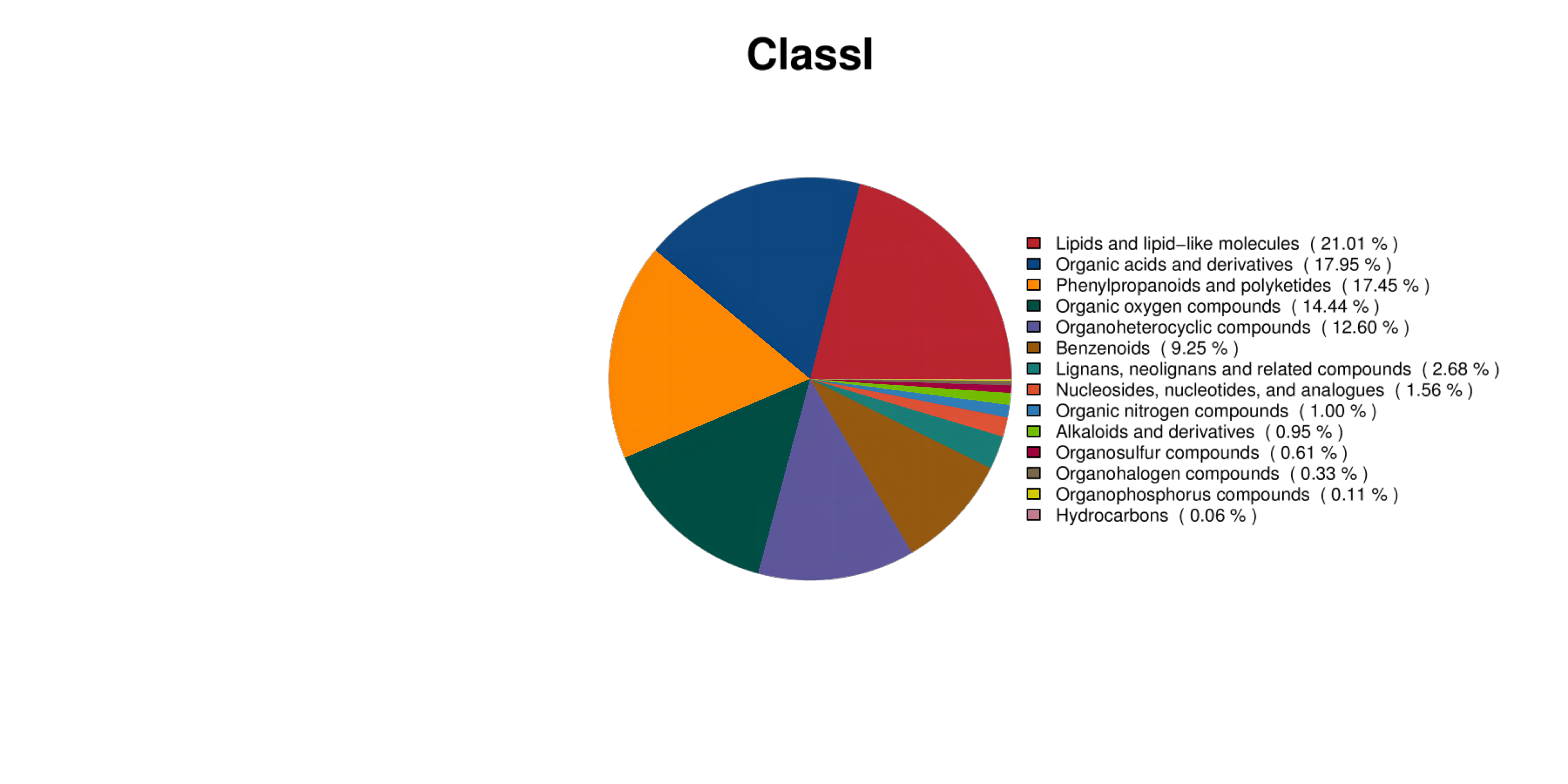

Supplement: Supplementary file 1 [file foods-14-03716-s001.zip › Figure S2.jpg]

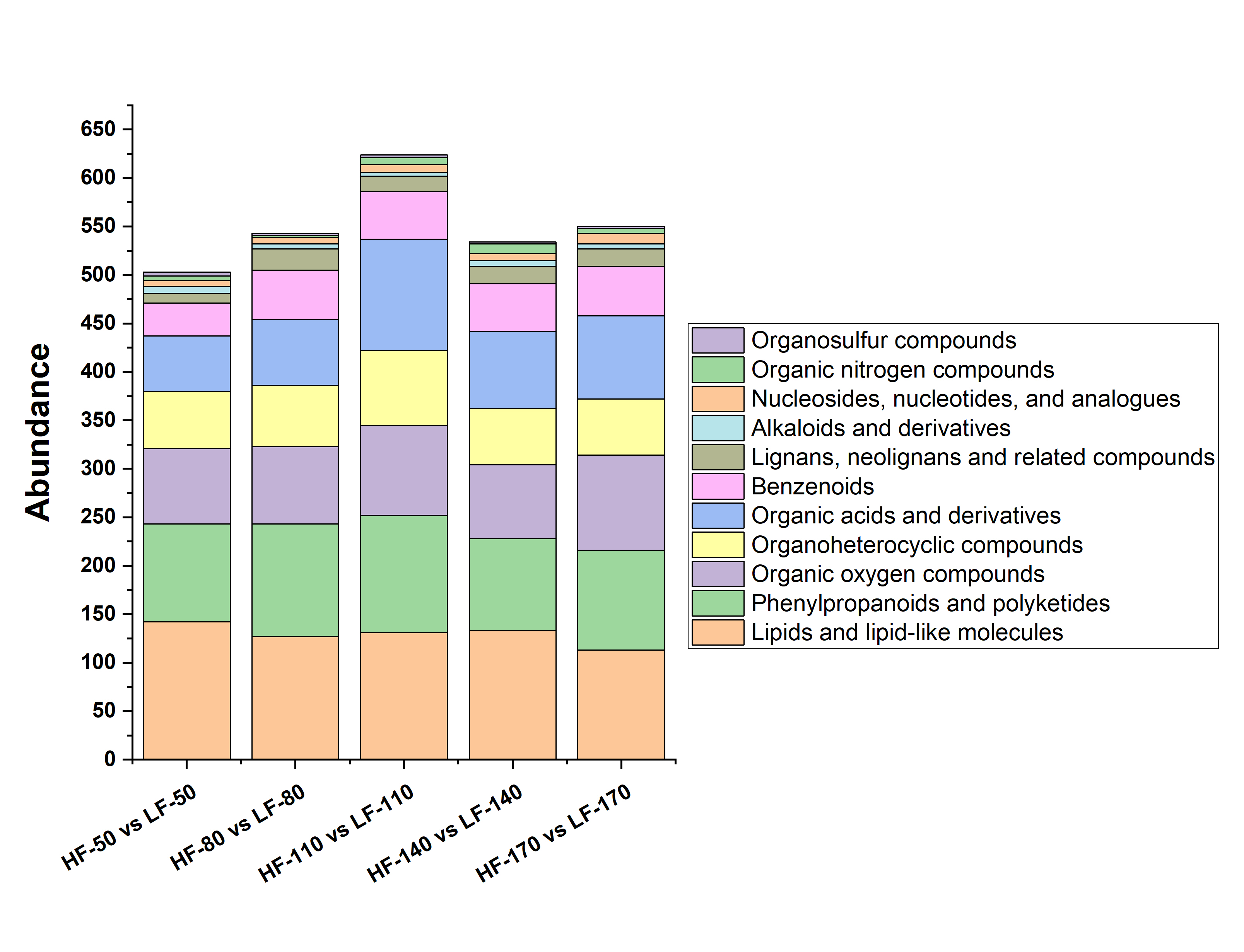

Supplement: Supplementary file 1 [file foods-14-03716-s001.zip › Figure S3.jpg]

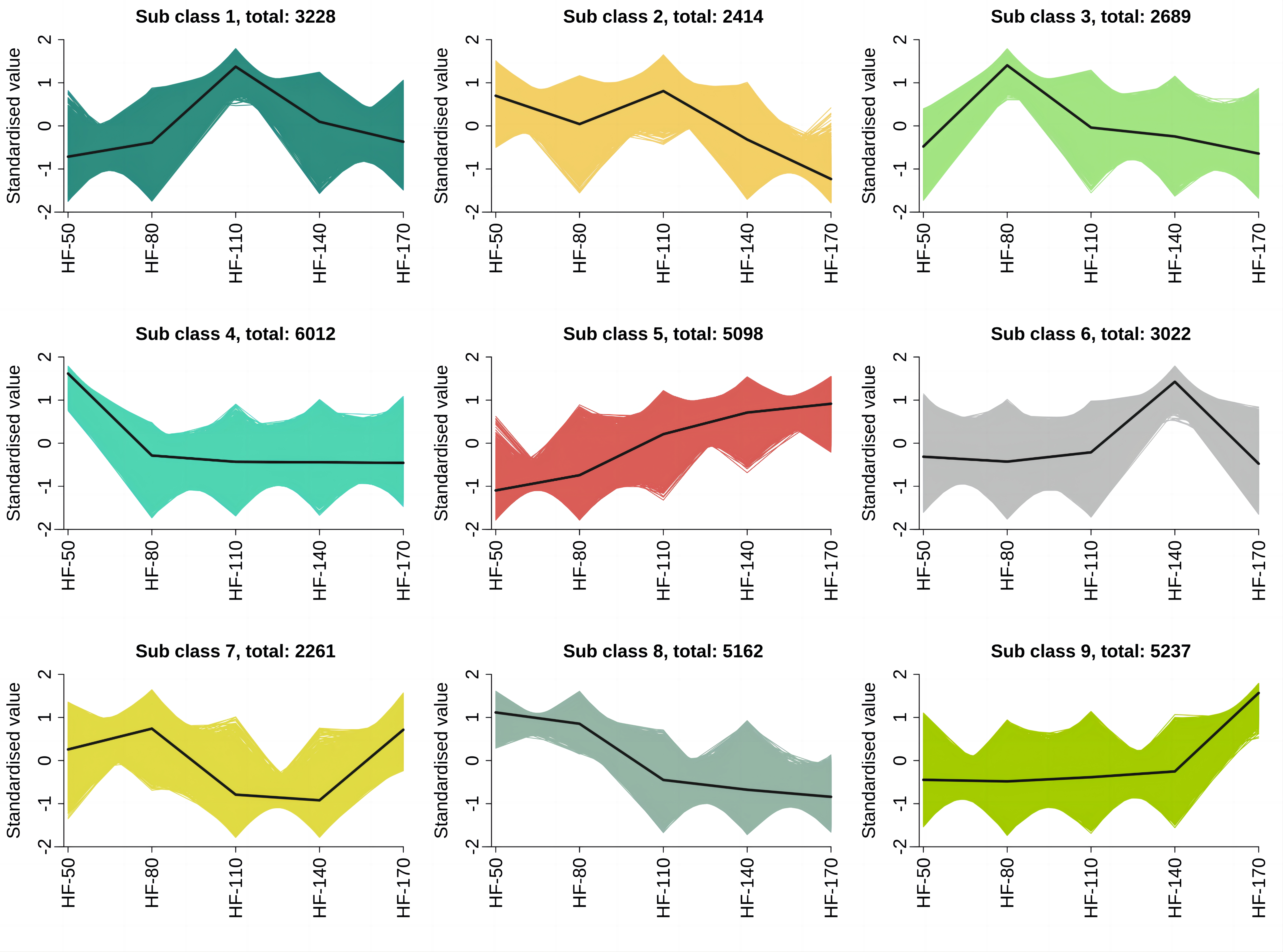

Supplement: Supplementary file 1 [file foods-14-03716-s001.zip › Figure S4.jpg]

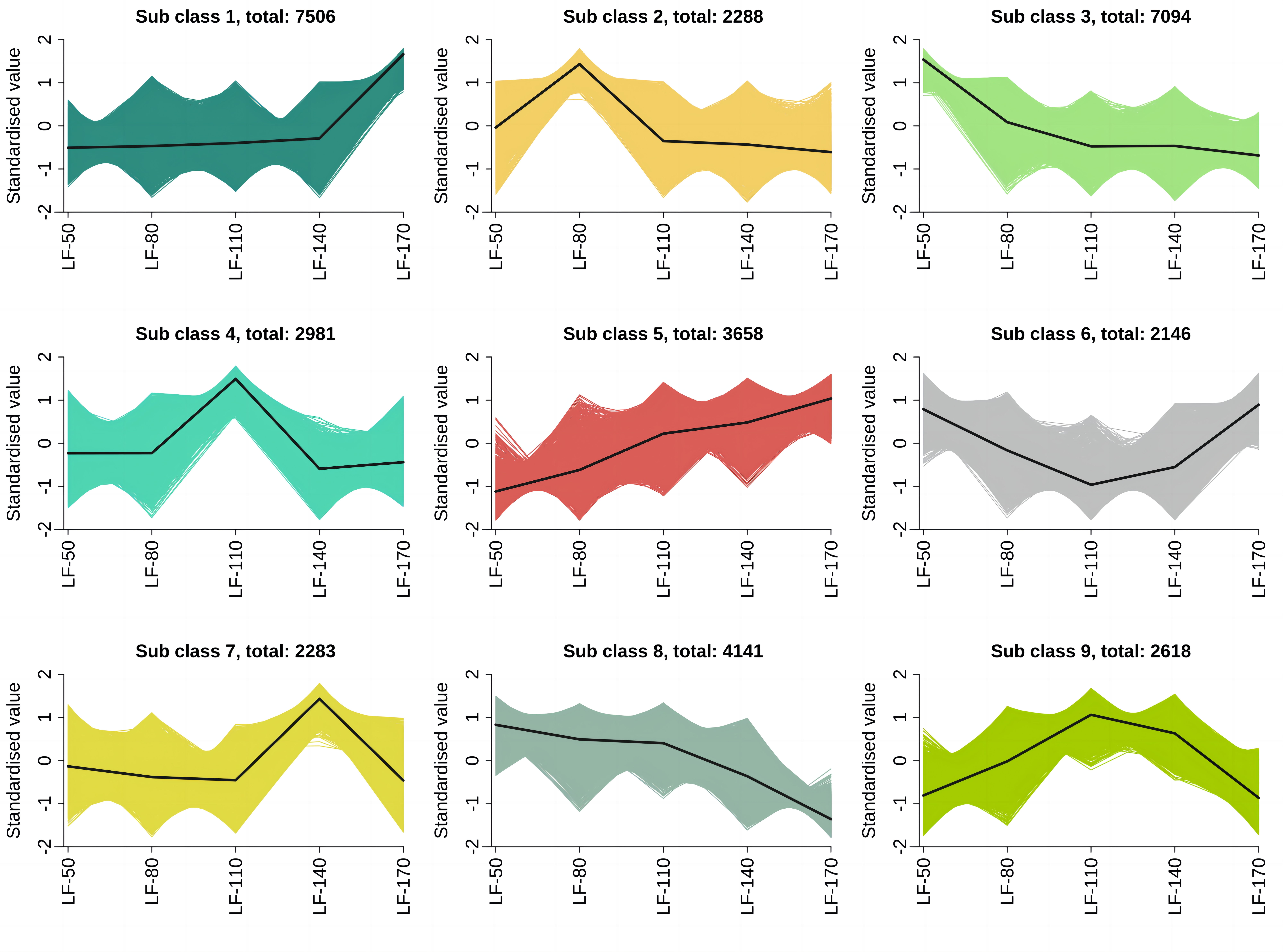

Supplement: Supplementary file 1 [file foods-14-03716-s001.zip › Figure S5.jpg]
